# A sensitive species-specific reverse transcription real-time PCR method for detection of *Plasmodium falciparum* and *Plasmodium vivax*

**DOI:** 10.1016/j.parepi.2017.04.001

**Published:** 2017-04-06

**Authors:** Kenneth Gavina, Eliana Arango, Catalina Alvarez Larrotta, Amanda Maestre, Stephanie K. Yanow

**Affiliations:** a Dept of Medical Microbiology and Immunology, University of Alberta, Edmonton, Alberta, Canada; b Grupo Salud y Comunidad, Universidad de Antioquia, Medellín, Colombia; c School of Public Health, University of Alberta, Edmonton, Alberta, Canada

**Keywords:** Malaria, Diagnostics, Surveillance, Species, *P. falciparum*, *P. vivax*, RT-qPCR

## Abstract

As the global burden of malaria decreases and countries strive towards disease elimination, there is a greater demand for sensitive diagnostics to target the submicroscopic reservoir of infection. We describe here a sensitive species-specific RT-qPCR method to differentiate between *Plasmodium falciparum* and *P. vivax* infections at the submicroscopic level. With amplification of the 18S rRNA genes from total nucleic acids (both DNA and RNA), we discern *P. falciparum* and *P. vivax* with a limit of detection of 10 parasites/mL and 18 copies/μL, respectively. This assay was validated with 519 blood samples, negative by thick-smear, from febrile and asymptomatic cohorts from Colombia. These results were directly compared to a qPCR-based method (DNA only) as the gold standard. Of the samples from patients who presented with fever (*n* = 274), 34 infections were identified by RT-qPCR (16 *P. falciparum*, 15 *P. vivax*, and 3 mixed), of which only 10 infections were identified at the species level by qPCR. Within the asymptomatic cohort (*n* = 245), 13 infections were identified by RT-qPCR (3 *P. falciparum*, 3 *P. vivax*, and 7 mixed), whereas the species for only one infection was determined by qPCR. We conclude that this species-specific RT-qPCR method provides a more sensitive tool for species identification compared to DNA based qPCR methods.

## 1 Introduction

Malaria continues to decline as a result of concerted control efforts and many countries are approaching disease elimination. According to the 2016 World Malaria Report (WHO, 2016a, WHO, 2016b), 17 countries have eliminated malaria, defined as the “reduction to zero indigenous cases for 3 or more years”, and six of these countries have been certified as malaria-free by WHO. The incidence of malaria is estimated to have decreased by > 40% globally between 2000 and 2015 (WHO, 2016a, WHO, 2016b). One of the final obstacles between elimination and eradication is the identification and treatment of individuals harbouring low-level parasites in the absence of clinical disease. These infections are often below the limit of detection of current field diagnostics (Okell et al., 2009). It is important to detect these infections, but also to identify the species in order to guide appropriate control measures and treatment. Twenty-one countries have the potential to eliminate local transmission of malaria by 2020, of which a majority of these countries had reported cases of both *P. falciparum* and *P. vivax* (WHO, 2016a, WHO, 2016b). Given the epidemiology of *Plasmodium* in these countries, there is a need for more sensitive tools to identify and determine the species of infections that contribute to ongoing disease transmission.

Although microscopy is the gold standard for diagnosis in the field, molecular diagnostics such as PCR and real-time quantitative PCR (qPCR) provide superior sensitivity and specificity and are used for epidemiological analyses within laboratory settings (Okell et al., 2009, Britton et al., 2016). There is no standard molecular diagnostic test for malaria and the reported methods use different approaches for sample processing, amplification, and detection (Roth et al., 2016). To maximize sensitivity, the majority of tests target multi-copy genes, including the *Plasmodium* 18S rRNA gene (Snounou et al., 1993), subtelomeric targets (Hofmann et al., 2015), and mitochondrial genes such as cytochrome *b* (Ekala et al., 2007), and *Plasmodium* mitochondrial cytochrome C oxidase III (Isozumi et al., 2015, Echeverry et al., 2016). One method concentrates large volumes of blood during the extraction process (Imwong et al., 2014). Another method uses photo-induced electron transfer fluorogenic primers to enhance detection (Talundzic et al., 2014). It was estimated that there are several thousand RNA copies of 18S rRNA per parasite (Murphy et al., 2012). Two methods that take advantage of these high copy numbers include RNA-specific nucleic acid based sequence amplification (NASBA) and reverse-transcriptase real-time PCR (RT-qPCR) (Schoone et al., 2000, Schneider et al., 2005, Hodgson et al., 2015). These molecular approaches result in superb sensitivity for detection of malaria parasites at the genus level, with reported limits of detection (LOD) as low as two parasites/mL (p/mL) (Kamau et al., 2011).

However, the sensitivities of tests to determine the species of *Plasmodium*, largely focused on *P. falciparum* and *P. vivax*, are much poorer, typically ranging from 127 to 4000 p/mL (Perandin et al., 2004, Rougemont et al., 2004, Shokoples et al., 2009, Kamau et al., 2013). Only two RT-qPCR based methods reported detection of *P. falciparum* and *P. vivax* at sensitivities equivalent to the genus-level assays (Wampfler et al., 2013, Adams et al., 2015). These tests demonstrated excellent sensitivity for species identification. These methods differ as one uses purified RNA as template for malaria species detection (Wampfler et al., 2013), while the method published by Adams et al. is based on detection of total parasite nucleic acid (both RNA and DNA), where RNA was reverse-transcribed and amplified by RT-qPCR.

In this study, we describe and validate a method to differentiate *P. falciparum* and *P. vivax* in submicroscopic infections that are positive at the genus level but below the sensitivity of current DNA-based species-specific assays. By using total nucleic acid as template, we demonstrate improved sensitivity and specificity for species differentiation compared to DNA-based methods. Furthermore, we compared RT-qPCR to qPCR in species detection of *Plasmodium* using patient samples.

## 2 Materials and methods

### 2.1 Patient samples

Ethical approval was obtained from the University of Alberta, Edmonton Canada, and the Universidad de Antioquia, Medellín, Colombia. Four groups of patient samples were used in this study: 1) a negative control group of samples obtained from the Alberta Provincial Laboratory for Public Health (ProvLab) (*n* = 25, negative by microscopy and qPCR); 2) blinded samples obtained from the ProvLab from travelers to malaria-endemic regions (*n* = 77, of which 17 samples were negative); 3) samples collected from febrile patients residing in a malaria-endemic district of Colombia (*n* = 274); and 4) samples from asymptomatic subjects residing in the same malaria-endemic district in Colombia (*n* = 245). Samples from febrile and asymptomatic participants in Colombia were collected between September 2013 and May 2016 as part of a larger study in the municipality of Puerto Libertador, Department of Cordoba, in Northwestern Colombia. The febrile cohort consisted of patients (aged 7–87 years) who presented to the malaria clinic in Puerto Libertador with suspected malaria and consented to participate in the study. The asymptomatic cohort included participants (aged 9–82 years) living in the villages who were enrolled as part of a community-based survey and consented to provide a blood sample to test for malaria.

Peripheral blood samples were collected by venipuncture in EDTA tubes. For assessment of microscopic malaria, thick smears were prepared from whole blood and stained with Field's stain. Slides were examined by an experienced microscopist. Samples were considered negative if no parasites were detected in 200 fields (1,000 × total magnification). Samples from Colombian participants that were positive by thick smear were excluded from this study. Whole blood was centrifuged and packed red blood cells (RBCs) were frozen and stored at − 20 °C for nucleic acid extraction.

### 2.2 Nucleic acid extraction and controls

Total nucleic acid was extracted from packed RBCs using a MagMAX 96 DNA Multi-Sample Kit (Applied Biosystem, Foster City, CA, USA) following the manufacturer's protocol with the following modifications: 130 μL of blood was extracted and eluted in the same volume, and a manual bench-top vortex was used in place of a plate shaker to minimize aerosol contamination. For the RNase experiment, RNase A (Thermofisher, USA) was added during the extraction procedure according to the manufacturer's protocol.

For *P. falciparum*, positive extraction controls were prepared from synchronized 3D7 ring-stage parasites cultured in human blood at a concentration of 10^6^ p/mL. In addition to cultured parasites, positive extraction controls for *P. falciparum* and *P. vivax* were prepared by cloning the respective 18S rRNA genes into a pGEM-T vector plasmid (Promega, Madison, WI, USA) and spiking the plasmid into uninfected blood. As *P. vivax* parasites are not sustainable in culture, the plasmid surrogate was used to determine the analytical sensitivity of our assay and reported as copies/μL. The *P. falciparum* plasmid surrogate was prepared and used to compare the analytical sensitivity with *P. falciparum* parasites. Negative blood was used as a no template control during extraction and carried through the workflow and PCR.

Strict precautions were followed to prevent cross-contamination and false positives including unidirectional workflow, minimal production of aerosols, and use of deep well plates. To this end, multiple positive and negative controls (minimum of six each) were included during extraction and carried throughout the workflow. In cases where controls failed, all samples on that plate were re-extracted from the original blood samples. Experiments were performed to test for cross-contamination between wells. Briefly, 96-well plates were run with 90 negative water samples and 6 positive controls (10^8^ p/mL *P. falciparum* 3D7 culture) randomly positioned on the plate and were extracted as previously described. Well-to-well contamination was not observed when samples were analyzed by qPCR and RT-qPCR.

### 2.3 qPCR

We used a published qPCR assay to detect *Plasmodium* at the genus level (Rougemont et al., 2004) and a modified assay developed in our laboratory to detect *P. falciparum* and *P. vivax* (Shokoples et al., 2009). This is the current protocol in use at the Alberta Provincial Laboratory for Public Health. Briefly, the assay was performed on an Applied Biosystems 7500 Fast Real-Time PCR System (Applied Biosystems, Foster City, CA, USA) with the following thermal profile: 15 minute activation step at 95 °C, 45 cycles of 15 seconds denaturation at 95 °C and one minute annealing/extension at 60 °C. For the reaction, 5 μL of template was added to a 20 μL reaction mix containing TaqMan Universal PCR Master Mix (Thermofisher, USA), 0.2 μM of primers, and 0.05 μM probe. The genus-level reaction uses primers and probes that target a region of the 18S rRNA gene that is conserved across all six species of *Plasmodium* (Rougemont et al., 2004) while the species-specific reaction targets an internal sequence that is variable across species (Shokoples et al., 2009). Samples with a cycle threshold (C_T_) ≤ 45 were considered positive. This C_T_ cut-off was determined based on previous work by our lab confirming *Plasmodium* positive samples with C_T_s of 40–45 by sequencing (Arango et al., 2013).

### 2.4 RT-qPCR

For *Plasmodium* species determination, RT-qPCR was performed on an ABI 7500 Fast Real-Time PCR System using the same species-specific *P. falciparum* and *P. vivax* primers and probes used for qPCR (Shokoples et al., 2009) and the following thermal profile: 20 s reverse transcription (RT) step at 50 °C, 20 s at 95 °C, 40 cycles of 3 s denaturation at 95 °C and 30 s annealing/extension at 60 °C. For the reaction, 5 μL of template was added to a 5 μL reaction mixture containing TaqMan Fast Virus 1-Step Master Mix (Thermofisher, USA), 0.8 μM of primers, and 0.2 μM probe. These primers were designed to target type A (asexual) rRNA genes on chromosomes five and seven for *P. falciparum* and chromosomes three and ten for *P. vivax.* The cut-off for the RT-qPCR reaction was set to C_T_ ≤ 40. Replicates of serially diluted positive controls were not consistently observed at a C_T_ > 38. Samples with a C_T_ between 38 and 40 were re-run in triplicate and called positive if two out of three replicates had C_T_ values ≤ 40. Only samples that were positive in both the genus assay and species assay were considered positive infections.

### 2.5 Reference materials

This protocol was validated following the Minimum Information for Publication of Quantitative qPCR Experiments (MIQE) guidelines using defined reference materials (Bustin et al., 2009). For standard curves, serial dilutions were prepared using *P. falciparum* 3D7 parasites from culture. Ring-synchronized parasites were quantified using a haemocytometer and serially diluted in human blood to final parasite concentrations of 10^5^, 10^4^, 10^3^, 10^2^, 10^1^, and 10^0^ p/mL and frozen prior to extraction. Serial dilutions of the *P. falciparum* and *P. vivax* plasmids ranged from 10^6^ to 10^0^ copies/μL. To measure the LOD, standards were run in three independent experiments with six replicates per dilution and repeated on three different days. As dilutions approached the LOD, three additional samples were run in replicates of six, totalling 36 replicates at 10 p/mL and 1 p/mL for the *P. falciparum* 3D7 parasites in blood, and 10 copies/μL and 1 copy/μL for the plasmid surrogates. The LOD was determined by probit analysis using MedCalc 16.4 (MedCalc Software, Ostend, Belgium). Negative blood samples included in this study were confirmed by microscopy and qPCR.

## 3 Results

### 3.1 Reaction efficiencies for *P. falciparum* and *P. vivax* RT-qPCR assays

The RT-qPCR reaction efficiency for the *P. falciparum* assay was 90.2%, based on serial dilutions of cultured parasites (R^2^ = 0.9975, slope of − 3.583). For the *P. vivax* assay, the reaction efficiency using the plasmid standard curve was 106.8% (R^2^ = 0.9984, slope of − 3.17). Non-specific amplification was not observed based on gel electrophoresis of amplified products.

### 3.2 Analytical sensitivity and specificity

**Fig. 1:**
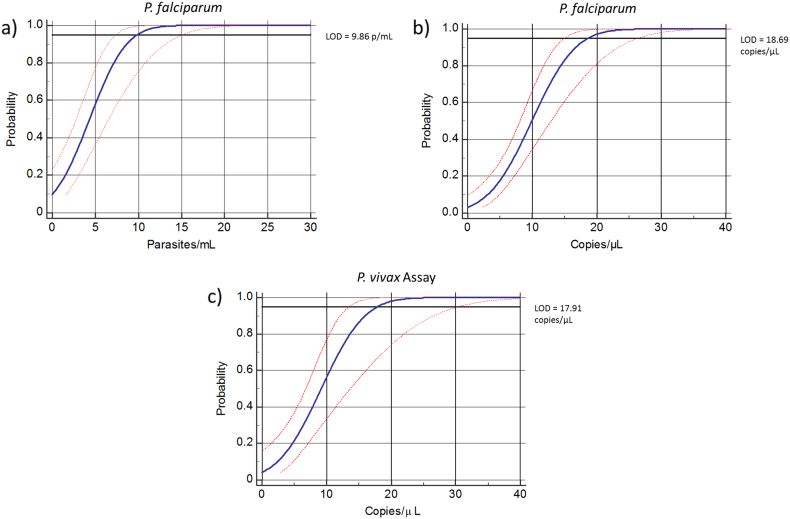
Analytical sensitivity determined by probit regression for a) *P. falciparum* assay (*P. falciparum* 3D7 malaria parasites), b) *P. falciparum* (plasmid surrogate) and c) *P. vivax* assay (plasmid surrogate). The blue line depicts the regression curve and the red dotted line represents the 95% confidence intervals.

Analytical sensitivity was determined from the dilution series described above (10^5^ to 10^0^ p/mL for *P. falciparum* and 10^6^ to 10^0^ copies/μL for *P. falciparum* and *P. vivax* plasmid surrogates), run in replicates of six in three independent experiments. Additional replicates were run as samples approached the LOD. The LOD with 95% confidence intervals derived from the *P. falciparum* standard was 10 p/mL (Fig. 1a), 19 copies/μL for the *P. falciparum* plasmid surrogate (Fig. 1b), and 18 copies/μL for the *P. vivax* plasmid surrogate (Fig. 1c). It should be noted that the plasmid surrogates used in this study served merely as positive controls for the lowest amount of nucleic acid template that can be detected for this target DNA.

We expected that the high sensitivity observed with the RT-qPCR assays was attributed to the increased copies of 18S rRNA transcript over genomic targets within the parasite (Murphy et al., 2012). To test this, we compared the C_T_ values for RT-qPCR performed on *P. falciparum* parasites (10^4^ p/mL) extracted with and without the addition of RNase A. Samples extracted without RNase had a mean C_T_ value of 22.81 ± 0.21, while samples extracted with the addition of RNase had a mean C_T_ value of 31.12 ± 0.16. We observed a significant increase in C_T_ for samples treated with RNase A (8.3 cycles greater; p < 0.0001, two-tailed paired *t*-test), consistent with just under ~ 1000 times more RNA target than gDNA template extracted from parasites.

Analytical specificity was assessed using *P. falciparum* 3D7 parasites and plasmid surrogate positive controls to test for potential cross-reactivity between the species-specific primers and probes. Thirty-six replicates (18 replicates of 10^6^ p/mL *P. falciparum* 3D7 control and 18 replicates of 10^6^ copies/μL *P. vivax* plasmid control) were analyzed in both assays. All 18 *P. falciparum* replicates were positive for *P. falciparum* and negative for *P. vivax* by RT-qPCR. Similarly, all 18 plasmid surrogate replicates were positive for *P. vivax* and negative for *P. falciparum* by RT-qPCR, giving an analytical specificity of 100%. Furthermore, 25 blood samples that were negative by microscopy and qPCR were assessed by RT-qPCR, and all samples were negative for *P. falciparum* and/or *P. vivax*.

### 3.3 Precision

Intra-assay variation (variation between replicates in the same experiment) and inter-assay variation (variation between replicates in different experiments) were assessed to determine the repeatability and reproducibility of the species-specific assays. Serial dilutions of positive controls (*P. falciparum* 10^5^ to 10^1^ p/mL and *P. vivax* 10^5^ to 10^1^ copies/μL) and three negative control samples were run in replicates of six across three different experiments performed on different days (Table 1). Precision analysis was performed on the basis of C_T_ values of the replicates and the coefficient of variation (%CV) was determined. Intra-assay %CV ranged from 0.01–0.03% and < 0.01–0.02% for the *P. falciparum* and *P. vivax* samples, respectively. Inter-assay %CV ranged from 0.01–0.26% and < 0.01–0.09% for *P. falciparum* and *P. vivax* samples, respectively.

**Table 1:** Reproducibility and repeatability of RT-qPCR assays.

Sample	Intra-assay mean (C_T_)	%CV^a^	Inter-assay mean (C_T_)	%CV^a^
*P.f.* 10^5^ p/mL	19.10	0.02	19.77	0.03
*P.f.* 10^4^ p/mL	22.33	0.03	22.83	0.26
*P.f.* 10^3^ p/mL	25.94	0.02	26.48	0.04
*P.f.* 10^2^ p/mL	29.43	0.01	29.38	0.01
*P.f.* 10^1^ p/mL	34.22	0.03	33.95	0.01
*P.v.* 10^5^ copies/μL	25.70	0.01	25.77	< 0.01
*P.v.* 10^4^ copies/μL	28.79	< 0.01	28.93	0.09
*P.v.* 10^3^ copies/μL	32.18	< 0.01	32.36	0.04
*P.v.* 10^2^ copies/μL	35.16	0.02	35.23	0.01
*P.v.* 10^1^ copies/μL	37.97	0.01	37.78	0.01
Negative	–	–	–	–
Negative	–	–	–	–
Negative	–	–	–	–

a %CV – coefficient of variation.

### 3.4 Sensitivity and specificity relative to qPCR

We directly compared species identification by RT-qPCR to qPCR using a dilution series of *P. falciparum* parasites (10^5^ to 10^0^ p/mL) to observe the differences in analytical sensitivity (Table 2). Using qPCR, we were able to detect 10^1^ p/mL (two of three replicates) with a mean C_T_ value of 38.49, whereas we detected 10^0^ p/mL (two of three replicates) when using RT-qPCR. Further, the C_T_ values at all dilutions were lower with RT-qPCR compared with qPCR.

**Table 2:** Direct comparison of RT-qPCR and qPCR for species identification using a serial dilution of *P. falciparum* 3D7 parasites.

Dilution	qPCR mean C_T_	RT-qPCR mean C_T_	p-Value^a^
10^5^ parasites/mL	23.29	18.79	< 0.0001
10^4^ parasites/mL	26.84	22.31	< 0.0001
10^3^ parasites/mL	30.43	25.95	< 0.0001
10^2^ parasites/mL	33.89	29.18	< 0.0001
10^1^ parasites/mL	38.49^c^	33.62	0.0046
10^0^ parasites/mL	ND^b^	39.54^c^	–

a Calculated by unpaired Student's *t*-test, statistically significant results (p < 0.05).

b Not detected.

c Only two of three replicates detected.

We next determined the clinical sensitivity and specificity of the RT-qPCR assay compared with qPCR using a blind panel of patient samples collected from the Alberta ProvLab (Table 3). Of 77 samples, 42 (54%) were identified by qPCR as *P. falciparum*, 18 (23%) as *P. vivax*, and 17 as negative. When analyzed by RT-qPCR, all 42 positives were correctly identified; however, an additional *P. falciparum* and one *P. vivax* sample were also identified by RT-qPCR as mixed infections and confirmed by sequencing. Based on qPCR as the gold standard, the diagnostic sensitivity for both RT-qPCR assays was 100%, while diagnostic specificities were 97.20% and 98.33% for the *P. falciparum* and *P. vivax* assays, respectively.

**Table 3:** Diagnostic sensitivity and specificity of RT-qPCR compared to qPCR performed on 77 clinical samples.

		qPCR^a^			
		*P. falciparum*		*P. vivax*	
		Positive	Negative	Positive	Negative
RT-qPCR	Positive	42	1	18	1
	Negative	0	35	0	59

		*P. falciparum* (%)	95% CI	*P. vivax* (%)	95% CI

Sensitivity		100	91.59–100	100	81.47–100
Specificity		97.20	85.47–99.93	98.33	91.06–99.96

a 17 of 77 samples were negative for both *P. falciparum* and *P. vivax* by qPCR.

### 3.5 Evaluation of submicroscopic infections in febrile and asymptomatic populations from Colombia

To determine whether RT-qPCR provides enhanced species identification of submicroscopic infections, samples collected from two cohorts in Colombia were analyzed by both qPCR and RT-qPCR (Table 4). One cohort included 274 participants who presented to the health facility with fever and suspected malaria. For the second cohort, samples were collected from 245 asymptomatic participants in community-based surveys. All samples from febrile and asymptomatic participants were negative by thick smear. In the febrile population, 52 samples were positive for *Plasmodium* (genus) DNA by qPCR. The species was identified for only 10 of these samples by qPCR compared with 34 samples analyzed by RT-qPCR. Similarly, 36 samples from the asymptomatic group were positive at the genus level by qPCR; the species could only be determined in one sample by qPCR, but 13 were identified at the species level by RT-qPCR. These results demonstrate that RT-qPCR outperformed qPCR for species identification in a field setting.

**Table 4:** qPCR and RT-qPCR analysis of clinical samples from participants from Colombia.

	Febrile population (*n* = 274)		Asymptomatic population (*n* = 245)	
	qPCR pos n (%^a^)	RT-qPCR pos n (%^a^)	qPCR pos n (%^a^)	RT-qPCR pos n (%^a^)
*Plasmodium* spp.	52 (19%)	–	36 (14.7%)	–
*P. falciparum*	4 (1.5%)	16 (5.8%)	0	3 (1.2%)
*P. vivax*	6 (2.2%)	15 (5.5%)	1 (< 0.1%)	3 (1.2%)
Mixed species^b^	0	3 (0.1%)	0	7 (2.9%)

a Percent positive in the study population.

b Positive for both *P. falciparum* and *P. vivax.*

## 4 Discussion

The ability to detect infections with low level parasitemia and the identification of the infecting species are two critical parameters guiding malaria treatment (CDC, 2013, WHO, 2015). In recent years, several methods have been developed to improve diagnostic sensitivity and specificity (Roth et al., 2016). One method used a high volume blood sample for extraction with a reported sensitivity as low as 20 p/mL, but identification was limited to the *Plasmodium* genus (Imwong et al., 2014). An RT-qPCR method was validated for the detection of *Plasmodium* 18S rRNA with a LOD of 2 p/mL; however, the sensitivity at the species level was 1200 p/mL for *P. falciparum* (Kamau et al., 2011). Another RT-qPCR method reported a species-specific sensitivity of < 16 p/mL and 19.7 copies/μL for *P. falciparum* and *P. vivax*, respectively (Adams et al., 2015). The LODs for this assay are among the lowest reported for malaria detection at the species level for both *P. falciparum* and *P. vivax*.

In this study we describe a sensitive method to differentiate submicroscopic malaria infections as low as 10 p/mL and 18 copies/μL for *P. falciparum* and *P. vivax*, respectively. This method is based on a previous protocol developed in our lab (Shokoples et al., 2009) that was further optimized for detection of RNA to take advantage of the high copy numbers of 18S rRNA transcript per parasite to increase assay sensitivity. One consideration that could be addressed in this assay is the sampling volume used at the point of extraction. By using only 130 μL of whole blood, our minimum theoretical LOD is 7–8 p/mL. As a single *P. falciparum* parasite can contain 3500–10,000 copies of 18S rRNA per parasite (Murphy et al., 2012), the LOD of 19 copies/μL determined by the *P. falciparum* plasmid surrogate corresponds to 19,000 copies/mL or 2–5 p/mL. This suggests that by increasing the sampling volume at the point of extraction, it is possible to further increase the sensitivity of this assay. The volume used here was selected to minimize potential cross-contamination during the high-throughput extraction process. While this assay provides excellent sensitivity for identification of malaria infections at the species level using well-controlled reference materials, due to the complex processing and equipment required, we believe that this method would require further improvement in order to be suitable for point-of-care testing.

While the specificity assessed with patient samples from ProvLab was high (97–98%), two mixed infections were detected by RT-qPCR but not by qPCR. These infections were confirmed by sequencing and can be explained by the improved sensitivity of the RT-qPCR compared with the qPCR species-specific assay. This was demonstrated with a direct comparison of RT-qPCR to qPCR using serial dilutions of parasites from culture. Furthermore, RT-qPCR was superior to qPCR for species identification with the patient samples from Colombia and detected 34/52 positive samples from the febrile cohort and 13/36 positive samples from the asymptomatic cohort.

However, 41 of the Colombian patient samples were positive at the genus level but the species was not identified. We first ruled out possible *P. malariae* infection since this species also co-circulates in Colombia; all samples were negative for this species by qPCR. There are several other explanations for the discrepancy between the genus and species detection. It is likely that the RNA was not sufficiently preserved in the field samples. RNA is highly labile and prone to degradation. Whole blood samples were collected from venipuncture and stored as frozen pellets prior to extraction. While the DNA may be preserved for detection by qPCR, the RNA template for species-identification may not. We tested this hypothesis on the discordant samples from our two Colombian cohorts (samples that were positive by genus screening but negative for species identification). For these samples, whole blood was pelleted and preserved in Trizol. Purified RNA was extracted, quantified by nano-drop (ND-1000, Thermo Scientific, USA), and screened by RT-qPCR. Of the 44 discordant samples, five additional samples were species identified (two *P. falciparum* and three *P. vivax*) when analyzed from RNA extracted from Trizol. Although the sample collection method in our study was not initially designed for analysis of RNA, these results suggest that RNA preservation methods at the point of sample collection may increase the sensitivity of our method, closer to what we observed with the reference materials.

Alternative explanations can also be considered. It is possible that these were mixed infections in which the concentration of template for each species was below the limit of detection for the species assay. This is consistent with our findings that the amplicons sequenced from a subset of these samples had overlapping electropherograms which could not be deciphered. Another possibility is that the target sequences for the primers and probes used in the species-specific assay are polymorphic in naturally circulating parasite populations. It is also possible that the genus reaction can detect non-*Plasmodium* sequences. Based on the published results of Rougemont et al. (2004), the genus probe cross-reacted with other 18S rRNA DNA from other pathogens (*Aspergillus, Toxoplasma, Neospora,* and Pneumocystis). However, the probes used in the species reactions were specific. This could account for a positive genus C_T_, but no species identification.

In conclusion, we present a species-specific RT-qPCR method for improved detection of low-level *P. falciparum* and *P. vivax* infections when compared directly to species detection by qPCR. This method could also be potentially adapted for field surveillance upon validation with sample collection methods used in the field (e.g., finger prick blood and preparation of dried blood spots) and appropriate preservation of RNA. Another application of this method could be for experimental human malaria infections in a controlled clinical environment. As molecular diagnostics are implemented more broadly for malaria surveillance, and particularly within the context of elimination (Britton et al., 2016), the increased sensitivity of this method for species identification could be important in epidemiological surveys and to define the submicroscopic burden of malaria.
